# Adaptive multi-parameter regularization approach to construct the distribution function of relaxation times

**DOI:** 10.1007/s13137-019-0138-2

**Published:** 2019-11-30

**Authors:** Mark Žic, Sergiy Pereverzyev, Vanja Subotić, Sergei Pereverzyev

**Affiliations:** 1 Ruđer Bošković Institute, P.O. Box 180, 10000 Zagreb, Croatia; 2 Department of Neuroradiology, Medical University of Innsbruck, Anichstrasse 35, 6020 Innsbruck, Austria; 3 Neuroimaging Research Core Facility, Medical University of Innsbruck, Anichstrasse 35, 6020 Innsbruck, Austria; 4 Institute of Thermal Engineering, Graz University of Technology, Inffeldgasse 25b, 8010 Graz, Austria; 5 Johann Radon Institute for Computational and Applied Mathematics, Altenbergerstrasse 69, 4040 Linz, Austria

**Keywords:** EIS, DFRT, Ill-posed problem, Regularization, 45F05, 65R30

## Abstract

Determination of the distribution function of relaxation times (DFRT) is an approach that gives us more detailed insight into system processes, which are not observable by simple electrochemical impedance spectroscopy (EIS) measurements. DFRT maps EIS data into a function containing the timescale characteristics of the system under consideration. The extraction of such characteristics from noisy EIS measurements can be described by Fredholm integral equation of the first kind that is known to be ill-posed and can be treated only with regularization techniques. Moreover, since only a finite number of EIS data may actually be obtained, the above-mentioned equation appears as after application of a collocation method that needs to be combined with the regularization. In the present study, we discuss how a regularized collocation of DFRT problem can be implemented such that all appearing quantities allow symbolic computations as sums of table integrals. The proposed implementation of the regularized collocation is treated as a multi-parameter regularization. Another contribution of the present work is the adjustment of the previously proposed multiple parameter choice strategy to the context of DFRT problem. The resulting strategy is based on the aggregation of all computed regularized approximants, and can be in principle used in synergy with other methods for solving DFRT problem. We also report the results from the experiments that apply the synthetic data showing that the proposed technique successfully reproduced known exact DFRT. The data obtained by our techniques is also compared to data obtained by well-known DFRT software (DRTtools).

## Introduction

In electrochemical impedance spectroscopy (EIS), the experiments are usually interpreted by fitting complex-valued impedance measurements $Z\left( {\omega_{j} } \right) = Z^{\prime } \left( {\omega_{j} } \right) + iZ^{\prime \prime } \left( {\omega_{j} } \right), j = 0,1, \ldots ,N - 1,$ against chosen equivalent electrical circuit (EEC) models. One of such EEC model is known as the Voight circuit (Barsoukov and Macdonald 2005) which is composed of a series of parallel capacitors $C_{m}$ and resistors $R_{m} , m = 0,1, \ldots ,M - 1$, for which the impedance can be written as:

1 $$ Z\left( {\omega_{j} } \right) = \mathop \sum \limits_{m = 0}^{M - 1} \frac{{R_{m} }}{{1 + i\omega_{j} \tau_{m} }}, \quad j = 0,1, \ldots ,N - 1, $$

where $\tau_{m} = R_{m} C_{m}$. The high-frequency cut-off resistance (*R*_*∞*_) can be also added to (1), but herein it is omitted for simplicity. This *R*_*∞*_ can be estimated from EIS measurements at large angular frequencies (*ω*).

Moreover, EIS experiments cannot often be described by a finite number of simple resistor–capacitor (RC) elements, because they involve distributed time constants. Then a Voigt circuit with an infinite number of RC elements can also be used to fit the impedance data $\left( {Z\left( {\omega_{j} } \right)} \right)$. However, instead of discrete values $R_{m} = \tau_{m} C_{m}^{ - 1}$ [see (1)], one should use $R = g\left( \tau \right)$ to obtain a continuous version of (1):

2 $$ Z\left( \omega \right) = \mathop \int \limits_{0}^{\infty } \frac{g\left( \tau \right)d\tau }{1 + i\omega \tau } , $$

where $g\left( \tau \right)$ describes the time relaxation characteristic of the electrochemical system under study.

Up to a certain extent $g\left( \tau \right)$ provides a circuit model-free representation of essential relaxation times, which are directly connected to the charge transfer process (see, e.g., Song and Bazant 2018). One should bear in mind that not only Voigt circuit, but also other known circuit models, such as a Cole–Cole (Cole and Cole 1941) model, Davidson–Cole (Davidson and Cole 1951) model, Warburg element (Barsoukov and Macdonald 2005), etc., can also be discussed in terms of the Eq. (2).

Furthermore, since we are interested in a real-valued solution $g\left( \tau \right)$ of (2), the Eq. (2) can be reformulated into the system of integral equations with operators $A_{1} ,A_{2}$:

3 $$ \left\{ {\begin{array}{*{20}l} {(A_{1} g)\left( \omega \right) = \mathop \int \limits_{0}^{\infty } \frac{g\left( \tau \right)d\tau }{{1 + \omega^{2} \tau^{2} }} = Z^{\prime } \left( \omega \right),    } \hfill \\ {(A_{2} g)\left( \omega \right) = \mathop \int \limits_{0}^{\infty } \frac{\omega \tau g\left( \tau \right)d\tau }{{1 + \omega^{2} \tau^{2} }} = - \,Z^{\prime \prime } \left( \omega \right),} \hfill \\ \end{array} } \right. $$

where $Z^{\prime } \left( \omega \right),  Z^{\prime \prime } \left( \omega \right)$ are real and imaginary parts of $Z\left( \omega \right)$.

If we observe that instead of the whole function $Z\left( \omega \right)$, only the impedance measurements $Z\left( {\omega_{j} } \right) = Z^{\prime } \left( {\omega_{j} } \right) + iZ^{\prime \prime } \left( {\omega_{j} } \right), j = 0,1, \ldots ,N - 1$ are available, then the system (3) is reduced to a *collocation* and can be abstractly written as:

4 $$ \begin{aligned} & T_{N} A_{1} g = T_{N} Z^{\prime } , \\ & T_{N} A_{2} g = - {\kern 1pt} \,T_{N} Z^{\prime \prime } , \\ \end{aligned} $$

where *T*_*N*_ is the so-called sampling operator, which assigns to each function *F*(*ω*) a vector of its values at the collocation points $\omega_{j}$, i.e., $T_{N} F = (F\left( {\omega_{0} } \right), F\left( {\omega_{1} } \right), \ldots ,F\left( {\omega_{N - 1} } \right).$

Recall that the collocation is a special form of discretization that arises when we replace the original problem, such as (3), by one in a finite dimensional space. In case of collocation this space is just the Euclidean space $R^{N}$ of vectors $u = \left( {u_{0} ,u_{1} , \ldots ,u_{N - 1} } \right)$, $v = \left( {v_{0} ,v_{1} , \ldots ,v_{N - 1} } \right)$ equipped with a scalar product

$$ \left\langle {u,v} \right\rangle_{{R^{N} }} = \mathop \sum \limits_{j = 0}^{N - 1} \gamma_{j} u_{j} v_{j} , $$

and the corresponding norm $\left\| \cdot \right\|_{{R^{N} }}$; here the weights $\gamma_{j} ,j = 0,1, \ldots .,N - 1$, are some positive numbers.

Note that if operators $A_{1} ,A_{2}$ are considered to be acting from the space $L_{2} \left( {0,\infty } \right)$ of real-valued square summable functions on $\left( {0,\infty } \right)$ then the Eq. (4), due to their finite dimension, are always solvable at least in the sense of least squares. Moreover, least square solutions of (4) can be reduced to the corresponding systems of *N* linear algebraic equations, such that no additional discretization is required and, as a result, no additional discretization error is introduced. Therefore, the impedance measurements considered as collocation data already hint at a way to approximate the solution of (2).

At the same time, in the EIS literature, one mainly finds two other different approaches for approximate solving of (2). In the first approach, which has been studied in Dion and Lasia (1999), Gavrilyuk et al. (2017) and Renaut et al. (2013), the integral operators $A_{1} ,A_{2}$ in (4) are additionally *discretized* by means of quadrature formulas. This approach can also subsume the methods (Boukamp 2015; Boukamp and Rolle 2017; Schichlein et al. 2002) in which the Eq. (2) is reduced to a deconvolution problem by a suitable change of variables, after which a numerical Fourier transform is employed. This procedure is usually conducted by using diverse approximation techniques such as quadrature formula (Boukamp 2015). The second approach, advocated in Saccoccio et al. (2014) and Wan et al. (2015), discretizes the operators $A_{1} ,A_{2}$ in (4) by projection onto the subspaces of piecewise linear or radial basis functions (RBFs).

In both previously mentioned approaches the level of additional discretization, governed by the number of knots of a quadrature formula or by the number of basis functions, should be properly tuned. Such tuning is especially crucial in the case of noisy impedance measurements when the application of regularization techniques avoids numerical instabilities in solving (4). Then, according to the Regularization theory (see, e.g., Mathe and Pereverzev 2003) the level of additional discretization of $A_{1} ,A_{2}$ in (4) should be coordinated with the amount of regularization. However, such coordination has not been discussed in the aforementioned literature yet. At the same time, this discretization issue does not even appear in (4) as no additional discretization of the operators $A_{1} ,A_{2}$ is introduced. Therefore, in the present paper, we study a new approach to obtain $g\left( \tau \right)$ that avoids any additional discretization of the operators in (4).

Furthermore, it is known that the imaginary and real components $Z^{\prime \prime } \left( {\omega_{j} } \right),  Z^{\prime } \left( {\omega_{j} } \right)$ of the impedance have different importance. A more thorough analysis of Eq. (3) indicates that $g\left( \tau \right)$ has a greater impact on $\text{Im} \left( {Z\left( \omega \right)} \right)$ then on $\text{Re} \left( {Z\left( \omega \right)} \right)$ (see, e.g., Dion and Lasia 1999). In that case it seems reasonable to treat the Eq. (4) with different amount of regularization (i.e., by applying two regularization parameters). At the same time, in the aforesaid literature, regularization of the Eq. (4) is governed by only *one* regularization parameter that does not allow a desired flexibility in exercising the regularization. There is one exception though, namely the paper (Zhang et al. 2016) that proposes to minimize a multi-parameter version of the Tikhonov regularization functional also over the values of regularization parameters. However, the above minimization problem may have several local minima, and one of them corresponds to zero values of the regularization parameters that leads to unregularized least-squares.

Herein, we propose a new approach that applies a multi-parameter regularization scheme without unnecessary additional discretization. We have also added to this approach an ability to automatically choose regularization parameter values. Note that this kind of endeavor has not been reported in the aforementioned literature. In addition, in order to enable an automatic regularization in the present study, we use the idea (Chen et al. 2015) of an aggregation of regularized solutions corresponding to different values of multiple regularization parameters.

## Multi-parameter regularization of the collocated impedance equations

In this section we analyze a methodology for joint regularization of the collocation Eq. (4) that leads to a multi-parameter regularization. The joint regularization can be formulated as the minimization of the objective functional:

5 $$ \varPhi \left( g \right): = \lambda_{1} \left\| {T_{N} A_{1} g - T_{N} Z^{\prime } } \right\|_{{R^{N} }}^{2} + \lambda_{2} \left\| {T_{N} A_{2} g + T_{N} Z^{\prime \prime } } \right\|_{{R^{N} }}^{2} + \left\| g \right\|_{{L_{2} \left( {0,\infty } \right)}}^{2} . $$

Here, the first two terms are the measures of data misfit that are weighed with the regularization parameters $\lambda_{1} ,\lambda_{2} \subseteq \left( {0,\infty } \right)$. These misfits measures are combined in (5) with a regularization measure. According to, e.g., Chen et al. (2015), the minimizer $g = g_{{\lambda_{1} ,\lambda_{2} }} \left( \tau \right)$ of (5), can be found from the operator equation:

6 $$ g + \lambda_{1} \left( {T_{N} A_{1} } \right)^{*} T_{N} A_{1} g + \lambda_{2} \left( {T_{N} A_{2} } \right)^{*} T_{N} A_{2} g = \lambda_{1} \left( {T_{N} A_{1} } \right)^{*} T_{N} Z^{\prime } - \lambda_{2} \left( {T_{N} A_{2} } \right)^{*} T_{N} Z^{\prime \prime } , $$

where $\left( {T_{N} A_{1} } \right)^{*}$ and $\left( {T_{N} A_{2} } \right)^{*}$ are the adjoins of $T_{N} A_{1}$ and $T_{N} A_{2}$ respectably, and they are defined by the relations:

7 $$ \langle u,T_{N} A_{1} f\rangle_{{R^{N} }} = \langle \left( {T_{N} A_{1} } \right)^{*} u,f\rangle_{{L_{2} \left( {0,\infty } \right)}} ,   \langle u,T_{N} A_{2} f\rangle_{{R^{N} }} = \langle \left( {T_{N} A_{2} } \right)^{*} u,f\rangle_{{L_{2} \left( {0,\infty } \right)}} , $$

that should be satisfied for any $f \subseteq L_{2} \left( {0,\infty } \right)$ and $u = \left( {u_{0} ,u_{1} , \ldots ,u_{N - 1} } \right) \subseteq R^{N} .$

In view of the definition of $T_{N} ,A_{1} {\mkern 1mu} {\text{and}}{\mkern 1mu} \left\langle {u,v} \right\rangle_{{R^{N} }} = \sum\nolimits_{j = 0}^{N - 1} {\gamma_{j} } u_{j} v_{j} ,\gamma_{j} > 0$ we have

$$ \begin{aligned} \langle u,T_{N} A_{1} f\rangle_{{R^{N} }} & = \mathop \sum \limits_{j = 0}^{N - 1} \gamma_{j} u_{j} \left( {\mathop \int \limits_{0}^{\infty } \frac{f\left( \tau \right)d\tau }{{1 + \omega_{j}^{2} \tau^{2} }}} \right) = \mathop \int \limits_{0}^{\infty } \left( {\mathop \sum \limits_{j = 0}^{N - 1} \frac{{\gamma_{j} u_{j} }}{{1 + \omega_{j}^{2} \tau^{2} }}} \right)f\left( \tau \right)d\tau \\ & = \left\langle {\mathop \sum \limits_{j = 0}^{N - 1} \frac{1}{{1 + \omega_{j}^{2} \tau^{2} }}\gamma_{j} u_{j} ,f} \right\rangle_{{L_{2} \left( {0,\infty } \right)}} . \\ \end{aligned} $$

Now from (7) we can conclude that for any $u = \left( {u_{0} ,u_{1} , \ldots ,u_{N - 1} } \right) \subseteq R^{N}$ it holds:

8 $$ \left( {T_{N} A_{1} } \right)^{*} u = \mathop \sum \limits_{j = 0}^{N - 1} \frac{1}{{1 + \omega_{j}^{2} \tau^{2}  }}\gamma_{j} u_{j} . $$

On the other hand, the definition of the adjoint operator $\left( {T_{N} A_{1} } \right)^{*}$ indicates that it should be *N*-dimensional operator from $R^{N}$ to $L_{2} \left( {0,\infty } \right)$ and, as such, it should allow the representation:

9 $$ \left( {T_{N} A_{1} } \right)^{*} \left( \cdot \right) = \mathop \sum \limits_{j = 0}^{N - 1} l_{i} \left( \tau \right)\langle e^{j} ,\cdot\rangle_{{R^{N} }} , $$

where $l_{j} \subseteq L_{2} \left( {0,\infty } \right),e^{j} \subseteq R^{N} .$ Comparing this with (8) we arrive at the formulas

10 $$ \begin{aligned} l_{j} \left( \tau \right) & = \frac{1}{{1 + \omega_{j}^{2} \tau^{2} }}, \\ e^{j} & = \left( {e_{0}^{j} ,e_{1}^{j} , \ldots ,e_{N - 1}^{j} } \right) \subseteq R^{N} , \\ e_{k}^{j} & = \delta_{kj} . \\ \end{aligned} $$

where $\delta_{kj}$ is the Kronecker delta, i.e., $\delta_{kj} = 0\,{\text{for}}\,k \ne j,\,{\text{and}}\,\delta_{jj} = 1$.

By a similar argument, we can also obtain the following representation:

11 $$ \left( {T_{N} A_{2} } \right)^{*} \left( \cdot \right) = \mathop \sum \limits_{j = N}^{2N - 1} l_{j} \left( \tau \right)\langle e^{j - N} ,\cdot\rangle_{{R^{N} }} , $$

where

12 $$ l_{j} \left( \tau \right) = \frac{{\omega_{j - N} \tau }}{{1 + \omega_{j - N}^{2} \tau^{2} }},\quad j = N,N + 1, \ldots ,2N - 1. $$

Next, from (9)–(12) one can deduce that the solution of (6) admits the representation:

13 $$ g_{{\lambda_{1} ,\lambda_{2} }} \left( \tau \right) = \mathop \sum \limits_{j = 0}^{N - 1} g_{j} \frac{1}{{1 + \omega_{j}^{2} \tau^{2} }} + \mathop \sum \limits_{j = N}^{2N - 1} g_{j} \frac{{\omega_{j - N} \tau }}{{1 + \omega_{j - N}^{2} \tau^{2} }}. $$

The unknown coefficients $g_{i} = 0,1, \ldots ,2N - 1,$ can be found from the following system of linear algebraic equations:

14 $$ \left\{ {\begin{array}{*{20}l} {g_{k} + \lambda_{1} \mathop \sum \limits_{j = 0}^{2N - 1} a_{1,k,j} g_{j} = \lambda_{1} \gamma  _{k} Z^{\prime } \left( {\omega_{k} } \right),} \hfill &\quad {k = 0,1, \ldots ,N - 1,} \hfill \\ {g_{k} + \lambda_{2} \mathop \sum \limits_{j = 0}^{2N - 1} a_{2,k,j} g_{j} = - \,\lambda_{2} \gamma  _{k - N} Z^{\prime \prime } \left( {\omega_{k - N} } \right),} \hfill &\quad { k = N,N + 1, \ldots ,2N - 1, } \hfill \\ \end{array} } \right._{ } $$

where $$ a_{1,k,N + k} = \frac{{\gamma_{k} }}{{2\omega_{k} }},\, k = 0,1, \ldots ,N - 1, $$.

$$ \begin{aligned} a_{1,k,j} & = \left\{ {\begin{array}{*{20}l} {\mathop \int \limits_{0}^{\infty   } \frac{{\gamma_{k} d\tau }}{{\left( {1 + \omega_{k}^{2} \tau^{2} } \right)\left( {1 + \omega_{j}^{2} \tau^{2} } \right)}}    = \frac{{\pi \gamma_{k} }}{{2\left( {\omega_{k} + \omega_{j} } \right)}} ,} \\ \quad \hfill {j = 0,1,2, \ldots, N - 1} \\ {\mathop \int \limits_{0}^{\infty   } \frac{{\gamma_{k }  \omega_{j - N} \tau d\tau }}{{\left( {1 + \omega_{k}^{2} \tau^{2} } \right)\left( {1 + \omega_{j - N}^{2} \tau^{2} } \right)}}_{ } = \frac{{\gamma_{k} \omega_{j - N} }}{{\left( {\omega_{k}^{2} - \omega^{2}_{j - N} } \right)}}\ln \left( {\frac{{\omega_{k} }}{{\omega_{j - N} }}} \right),} \hfill \\ \quad {j = N, \ldots ,2N - 1;\quad j \ne N + k,} \hfill \\ \end{array} } \right. \\ a_{2,k,k - N} & = \frac{{\gamma_{k - N} }}{{2\omega_{k - N} }}, \quad k = N,N + 1, \ldots ,2N - 1, \\ a_{2,k,j} & = \left\{ {\begin{array}{*{20}l} {\mathop \int \limits_{0}^{\infty   } \frac{{\gamma_{k - N}  \omega_{k - N} \tau d\tau }}{{\left( {1 + \omega_{k - N}^{2} \tau^{2} } \right)\left( {1 + \omega_{j}^{2} \tau^{2} } \right)}}_{ }    =    \frac{{\gamma_{k - N} \omega_{k - N} }}{{\left( {\omega_{k - N}^{2} - \omega_{j}^{2} } \right)}}\ln \left( {\frac{{\omega_{k - N} }}{{\omega_{j} }}} \right),} \hfill \\ { j = 0,1,2, \ldots ,N - 1;\quad j \ne k - N,} \hfill \\ {\mathop \int \limits_{0}^{\infty   } \frac{{\omega_{k - N}^{ } \omega_{j - N} \gamma_{k - N } \tau^{2} d\tau }}{{\left( {1 + \omega_{k - N}^{2} \tau^{2} } \right)\left( {1 + \omega_{j - N}^{2} \tau^{2} } \right)}}_{ } =     \frac{\pi }{2}\gamma_{k - N} \frac{1}{{\omega_{k - N} \omega_{j - N } \left( {\omega_{k - N} + \omega_{j - N} } \right)}},} \hfill \\ \quad {j = N,N + 1, \ldots ,2N - 1,} \hfill \\ \end{array} } \right. \\ \end{aligned} $$

and following (Zoltowski 1984) we use the following weights

15 $$ \gamma_{k} = \left| {Z\left( {\omega_{k} } \right)} \right|^{ - 2} , k = 0,1, \ldots ,N - 1, $$

in the definition of the data misfits norm $\left\| \cdot \right\|_{{R^{N} }}$.

From (13), (14) it is clear that for given $\lambda_{1} ,\lambda_{2}$ the regularized approximate solution $g_{{\lambda_{1} ,\lambda_{2} }}$ of (4) can be constructed without additional discretization of the integral operators $A_{1} , A_{2}$.

## Aggregation of the regularized approximants in weighted norms

Note that in the EIS literature, the weighted solutions $\tau^{\nu } g\left( \tau \right),  \nu = 1,$ of the impedance Eq. (2) are of interest by themselves (see, e.g., Dion and Lasia 1999; Wan et al. 2015) and they are often called distribution functions of relaxation time (DFRT). Moreover, researchers are often interested in the behavior of DFRT only within a specific time window $\tau \subseteq \left[ {W_{min} , W_{max} } \right] \subset \left[ {0,\infty } \right)$. Therefore, for example, if $\tilde{g}\left( \tau \right)$ is an approximate solution of the impedance Eq. (2), such as $\tilde{g} \left( \tau \right) = g_{{\lambda_{1} ,\lambda_{2} }} \left( \tau \right)$, then it seems to be reasonable to measure approximation error in the weighted norm:

$$ \left\| {g - \tilde{g}} \right\|_{{L_{2,\nu } \left( {W_{min} , W_{max} } \right)}} = \left\{ { \mathop \int \limits_{{W_{min} }}^{{W_{max} }} \left[ {\tau^{\nu }  g\left( \tau \right) - \tau^{\nu } \tilde{g}\left( \tau \right)} \right]^{2} d\tau   } \right\}^{{\frac{1}{2}}} . $$

It is clear that this norm is a Hilbert space norm which is generated by the scalar product:

$$ \langle f,g\rangle_{{L_{2,\nu } \left( {W_{min} , W_{max} } \right)}} = \mathop \int \limits_{{W_{min} }}^{{W_{max} }} \tau^{2\nu } g\left( \tau \right)f\left( \tau \right)d\tau . $$

While we have described the explicit procedure (13), (14) for approximating the solutions of (2) directly from the impedance measurements without the application of discretization, there is still a question about the choice of the regularization parameters $\lambda_{1} ,\lambda_{2}$ that determines suitable relative weighting between these measurements. By setting $\lambda_{1} = 0\,{\text{or}}\,\lambda_{2} = 0$, one may reduce this question to the case discussed in Gavrilyuk et al. (2017). In another particular case $\lambda_{1} = \lambda_{2} = \lambda$, one may choose a suitable value of $\alpha = \lambda^{ - 1}$ by a cross-validation technique, as it was suggested in Wan et al. (2015). In both particular situations, one in fact deals with a single-parameter regularization which is applied in, e.g., Weese (1992) FTIKREG and DRTtools (Wan et al. 2015) software. However, a multi-parameter regularization is much less studied in EIS topic, especially when multiple regularization parameters are employed to construct a common misfit measure as in (5). Herein, we use new findings developed originally for inverse problems of satellite geodesy (Chen et al. 2015) and recently adjusted in the context of EIS (Zic and Pereverzyev 2018).

Note that known regularization parameter choice strategies usually consist of using some criteria for selecting only one particular candidate from a family of approximate solutions calculated for different values of regularization parameters from a sufficiently wide range. In contrast, in Chen et al. (2015) it is proposed to construct a new approximate solution in the form of a linear combination of all calculated approximants. In the present context this means, for example, that at first we calculate $g_{{\lambda_{1} ,\lambda_{2} }} \left( \tau \right)$ for some values $\lambda_{1} = \lambda_{1,p} ,  p = 0,1,2, \ldots ,P - 1,  \lambda_{2} = \lambda_{2,q} ,q = 0,1,2, \ldots ,Q - 1,$ deserving consideration, and then consider a new approximate solution:

16 $$ \tilde{g}\left( \tau \right) = \mathop \sum \limits_{m = 0}^{PQ - 1} c_{m} g^{m} \left( \tau \right), $$

where

$$ \begin{aligned} &  g^{m} \left( \tau \right) = g_{{\lambda_{1,p} ,\lambda_{2,q} }} \left( \tau \right), \\   & m = Pq + p,\quad  p = 0,1,2, \ldots ,P - 1, \quad q = 0,1,2, \ldots ,Q - 1, \\   & m = 0,1,2, \ldots ,PQ - 1, \\ \end{aligned} $$

and $c_{m}$ are coefficients to be determined.

In view of the previously mentioned discussion, it is natural to choose the coefficients *c*_*m*_ such that $\tilde{g}$ provides the best approximation of the exact solution $g$ in the norm $\left\| \cdot \right\|_{{L_{2,\nu } \left( {W_{min} W_{max} } \right)}}$ among all linear combinations (16). Since $\left\| \cdot \right\|_{{L_{2,\nu } \left( {W_{min} W_{max} } \right)}}$ is a Hilbert space norm, then the coefficients vector $\vec{c} = \left( {c_{0} ,c_{1} , \ldots ,c_{PQ - 1} } \right)$ corresponding to the best approximation (16) should solve the following matrix vector equation:

17 $$ G\overrightarrow {c } = \vec{F}, $$

where

$$ \begin{aligned} \vec{F} & = \left( {F_{m} } \right)_{m = 0}^{PQ - 1} , \\ F_{m} & = \langle g,g^{m} \rangle_{{L_{2,\nu } \left( {W_{min} ,W_{max} } \right)}} = \mathop \int \limits_{{W_{min} }}^{{W_{max} }} \tau^{2\nu } g\left( \tau \right)g^{m} \left( \tau \right)d\tau , \\ G & = \left( {G_{m,n} } \right)_{m,n = 0}^{PQ - 1} , \\ G_{m,n} & = \langle g^{m} ,g^{n} \rangle_{{L_{2,\nu } \left( {W_{min} , W_{max} } \right)}} = \mathop \int \limits_{{W_{min} }}^{{W_{max} }} \tau^{2\nu } g^{m} \left( \tau \right)g^{n} \left( \tau \right)d\tau . \\ \end{aligned} $$

If the regularized approximants

18 $$ g^{m} \left( \tau \right) = g_{{\lambda_{1,p} ,\lambda_{2,q} }} \left( \tau \right) = \mathop \sum \limits_{j = 0}^{2N - 1} g_{j}^{m} l_{j} \left( \tau \right), $$

are already calculated from (13), (14), then the elements of the matrix $G$ can be exactly calculated as well. Indeed, from (18) it follows that

$$ G_{m,n} = \mathop \sum \limits_{i = 0}^{2N - 1} \mathop \sum \limits_{i = 0}^{2N - 1} g_{i}^{m} g_{j}^{n} b_{i,j} , $$

where

$$ b_{ij} = \left\langle {l_{i} ,l_{j} } \right\rangle_{{L_{2,\nu } \left( {W_{min} ,W_{max} } \right)}} = \mathop \int \limits_{{W_{min} }}^{{W_{max} }} \tau^{2\nu } l_{i} \left( \tau \right)l_{j} \left( \tau \right)d\tau $$

and $l_{i} \left( \tau \right),l_{j} \left( \tau \right)$ are given by (10), (12). The above integrals can be explicitly^1^ calculated. For example, for $\nu = 1\,{\text{and}}\,i,j = N + 1, \ldots ,2N - 1$ we have

$$ b_{ij} = \left\{ {\begin{array}{*{20}l} {\left. {\frac{{W_{max} - W_{min} }}{{\omega_{i - N} \omega_{j - N} }} + \frac{{\omega_{j - N}^{3} \tan^{ - 1} \left( {\omega_{i - N} \tau } \right) - \omega_{i - N}^{3} \tan^{ - 1} \left( {\omega_{j - N} \tau } \right)}}{{\omega_{i - N}^{2} \omega_{j - N}^{2} \left( {\omega_{i - N}^{2} - \omega_{j - N}^{2} } \right)}}} \right|_{{\tau = W_{min} }}^{{\tau = W_{max} }} ,} \hfill & {i \ne j} \hfill \\ {\left. {\frac{{\omega_{i - N} \tau \left( {2 + \left( {1 + \omega_{i - N}^{2} \tau^{2} } \right)^{ - 1} } \right) - 3\tan^{ - 1} \left( {\omega_{i - N } \tau } \right)}}{{2\omega_{i - N}^{3} }}} \right|_{{\tau = W_{min} }}^{{\tau = W_{max} }} ,} \hfill & {i = j.} \hfill \\ \end{array} } \right. $$

On the other hand, the components $F_{m}$ of the vector $\vec{F}$ in (17) depend on the unknown solution $g$ of (2), and therefore are inaccessible.

At the same time, in Chen et al. (2015) and Kindermann et al. (2018) we can find an approach to estimate the components of the vector $\vec{F}$ by using the so-called quasi-optimality criterion in the linear functional strategy. The advantage of this approach is that the values of the scalar products $F_{m} = \left\langle {g,g^{m} } \right\rangle_{{L_{{2,\nu \left( {W_{min} ,  W_{max} } \right)}} }}$ of the solution $g$ can be estimated much more accurately than the solution $g$ in the norm $\left\| \cdot \right\|_{{L_{2,\nu } \left( {W_{min} W_{max} } \right)}}$. According to Chen et al. (2015) and Kindermann et al. (2018) we estimate the scalar product $\left\langle {g,g^{m} } \right\rangle$ by $\left\langle {f_{\alpha } ,g^{m} } \right\rangle_{{L_{{2,\nu \left( {W_{min} ,  W_{max} } \right)}} }}$, where $f_{\alpha } \left( \tau \right)$ is the regularized approximate solutions $g_{{\lambda_{1} ,\lambda_{2} }} \left( \tau \right)$ given by (13) and constructed for $\lambda_{1} = 0, \lambda_{2} = \alpha$, with the use of only imaginary part of the impedance data. The reason for this is that the imaginary part may allow better accuracy than the real part of the impedance (see, e.g., Dion and Lasia 1999).

In principle, the values of $\lambda_{2} = \alpha$ may be taken from the same set $\left\{ {\lambda_{2,q} } \right\}_{q = 0}^{Q - 1}$ as the one above. However, in Chen et al. (2015) and Kindermann et al. (2018) it is suggested to take $\alpha = \alpha_{s}$ from a geometric sequence $\alpha_{s} = \alpha_{0} \beta^{s} , s = 0,1, \ldots ,S - 1,   S > Q$. Therefore, we consider

$$ f_{{\alpha_{s} }} \left( \tau \right) = \mathop \sum \limits_{j - N}^{2N - 1} g_{j}^{0,s} l_{j} \left( \tau \right), $$

where $g_{j} = g_{j}^{0,s}$ are the solutions of the linear system (14) with $\lambda_{1} = 0, \lambda_{2} = \alpha_{s} , s = 0,1, \ldots ,S - 1$, and

$$ \left\langle {f_{{\alpha_{s} }} ,g^{m} } \right\rangle_{{L_{2,\tau } \left( {W_{min} ,W_{max} } \right)}} = \mathop \sum \limits_{i = N}^{2N - 1} \mathop \sum \limits_{j = 0}^{2N - 1} g_{i}^{0,s} g_{j}^{m} b_{i,j} . $$

Then, according to the quasi-optimality criterion in the linear functional strategy (Kindermann et al. 2018) we select such $s = s_{m}$ that

$$ \begin{aligned} & \left| {\left\langle {f_{{\alpha_{{s_{m} }} }} ,g^{m} } \right\rangle_{{L_{2,\nu } \left( {W_{min} , W_{max} } \right)}} - \left\langle {f_{{\alpha_{{s_{m} }} - 1}} ,g^{m} } \right\rangle_{{L_{2,\nu } \left( {W_{min} , W_{max} } \right)}} } \right| \\ & \quad = min\left\{ {\left| {f_{{\alpha_{s} }} ,g^{m}_{{L_{2,\nu } \left( {W_{min} , W_{max} } \right)}} - f_{{\alpha_{s - 1} }} ,\quad g^{m}_{{L_{2,\nu } \left( {W_{min} , W_{max} } \right)}} } \right|, \quad s = 1,2, \ldots ,S - 1} \right\}. \\ \end{aligned} $$

The theoretical analysis of Kindermann et al. (2018) guarantees that the values of $F_{m} = \left\langle {g,g^{m} } \right\rangle$ are well approximated by the values of $\tilde{F}_{m} = \left\langle {f_{{\alpha_{sm} }} ,g^{m} } \right\rangle$.

Recall that vector $\vec{c}$ of the coefficients of the best linear combination of (16) approximating the solution $g$ of (2) in the norm $\left\| \cdot \right\|_{{L_{2,\nu } \left( {W_{min} W_{max} } \right)}}$ should solve the matrix vector Eq. (17).

According to the aggregation strategy (Chen et al. 2015) we approximate this ideal vector by the vector $\tilde{c} = \left( {\tilde{c}_{0} ,\tilde{c}_{1, \ldots ,} \tilde{c}_{PQ - 1} } \right)$ solving in the least squares sense the approximate matrix vector equation

$$ G\tilde{c} = \tilde{F}, $$

19 $$ \tilde{F} = \left( {\tilde{F}_{0} ,\tilde{F}_{1} , \ldots ,\tilde{F}_{PQ - 1} } \right). $$

Then the theoretical analysis of Chen et al. (2015) guarantees that the approximate solution

20 $$ \tilde{g}\left( \tau \right) = \mathop \sum \limits_{m = 0}^{PQ - 1} \tilde{c}_{m} g^{m} \left( \tau \right) = \mathop \sum \limits_{m = 0}^{PQ - 1} \mathop \sum \limits_{j = 0}^{N - 1} \tilde{c}_{m} g_{j}^{m} \frac{1}{{1 + \omega_{j}^{2} \tau^{2} }} + \mathop \sum \limits_{m = 0}^{PQ - 1} \mathop \sum \limits_{j = N}^{2N - 1} \tilde{c}_{m} g_{j}^{m} \frac{{\omega_{j - N} \tau }}{{1 + \omega_{j - N}^{2} \tau^{2} }}, $$

is almost as good as the best linear approximation (16) of the calculated regularized solutions $g_{{\lambda_{1,p} ,\lambda_{2,q} }} \left( \tau \right)$.

We have described an adaptive procedure that automatically constructs an approximate solution of (2). This procedure should theoretically perform at the level of the best regularized approximant $g_{{\lambda_{1} ,\lambda_{2} }} \left( \tau \right)$ calculated according to (13), (14) for a given range of $\lambda_{1} , \lambda_{2}$. The input of the procedure consists of the impedance data $Z\left( {\omega_{j} } \right),  j = 0,1, \ldots ,N - 1$, the weights $\gamma_{j} ,  j = 0,1, \ldots ,N - 1$ determining the misfits measures, the endpoints $W_{min} , W_{max}$ of the time window of interest, and the numbers $\lambda_{0,1} ,\lambda_{0,2} ,\alpha_{0} ,P,Q,S$ defining the range of the regularization parameters under consideration.

## Experimental

### Synthetic and polluted ZARC2 and FRAC2 impedance data

The synthetic data in this work were prepared by using both Cole–Cole (i.e., ZARC) and Davidson–Cole (i.e., FRAC) relations. According to the literature (Barsoukov and Macdonald 2005), ZARC is widely used in Distribution Function of Relaxation Times (DFRT) study (see, e.g., Wan et al. 2015); and thus, a model of two ZARC elements was used to compute the synthetic ZARC2 data in this work:

21 $$ Z_{synth} \left( \omega \right) = R_{\infty } + \frac{{R_{1} }}{{1 + \left( {i\omega \tau_{0,1} } \right)^{{n_{1} }} }} + \frac{{R_{2} }}{{1 + \left( {i\omega \tau_{0,2} } \right)^{{n_{2} }} }}, $$

where $\omega$ is angular frequency, $R_{\infty } , R_{j} , j = 1, 2$ are resistances and $\tau_{0,j}$ is a time constant.

On the other hand, FRAC element is not often seen in the real experiments although it has been commonly applied when testing different DFRT approaches (Boukamp and Rolle 2017; Dion and Lasia 1999). This element is of a special interest in our paper as it is very hard to reconstruct DFRT data due to discontinuities at some time points $\tau$. Hence, to provide a more complete study, a model of two FRAC elements was used to prepare the synthetic FRAC2 data:

22 $$ Z_{\text{synth}} \left( \omega \right) = R_{\infty } + \frac{{R_{1} }}{{\left( {1 + i\omega \tau_{0,1} } \right)^{{n_{1} }} }} + \frac{{R_{2} }}{{\left( {1 + i\omega \tau_{0,2} } \right)^{{n_{2} }} }}. $$

Herein, we are interested in the comparison of performances of different regularization techniques, and we consider the synthetic ZARC2 and FRAC2 data polluted by noise:

23 $$ Z_{poll} \left( {\omega_{j} } \right) = Z_{synt} \left( {\omega_{j} } \right) \cdot \left( {1 + NF \cdot \left( {\eta_{j}^{{\prime }} + i\eta_{j}^{{\prime \prime }} } \right)} \right), $$

where *NF* = 0.001 and $\eta^{\prime } ,\eta^{\prime \prime }$ are independent normally distributed random variables with zero mean and the variance one. This approach to simulate noisy data is similar to one reported in Wan et al. (2015).

As the majority of regularization techniques in EIS study employs discretization, the applied frequency values were equally spaced in the logarithmic scale from 0.01 Hz to 100 kHz, taking 10 points per decade. Parameters used to compute the synthetic ZARC2 and FRAC2 data are given in Table 1.

**Table 1:** Parameters used to compute the synthetic ZARC2 and FRAC2 data

Synthetic data	$R_{\infty }$ (Ω cm^2^)	*R*_1_ (Ω cm^2^)	$\tau_{0,1}$ (s)	*n*_1_	*R*_2_ (Ω cm^2^)	$\tau_{0,2}$ (s)	*n*_2_
ZARC2/FRAC2	10	50	0.01	0.7	50	0.001	0.7

### Analytical DFRT for ZARC2 and FRAC2

Since we applied synthetic ZARC2 and FRAC2 data, it is natural to compare their reconstructed DFRT with the analytical ones. This is a usual approach which enables fast and consistent evaluation of new DFRT methods (see, e.g., Dion and Lasia 1999; Wan et al. 2015). This approach is especially relevant as our reconstruction method (i.e., DFRT-Py) utilizes automatic choice of parameters. Herein, we applied the following analytical form to compute DFRT (abbreviated here as DFRT_ZARC2_) of the synthetic ZACR2 data:

24 $$ g\left( \tau \right) = \frac{{R_{1} }}{{2\uppi\tau }}\frac{{\sin \left( {\left( {1 - n_{1} } \right)\pi } \right)}}{{\cosh \left( {n_{1} \ln \left( {\frac{\tau }{{\tau_{0,1} }}} \right)} \right) - \cos \left( {\left( {1 - n_{1} } \right)\uppi} \right)}} + \frac{{R_{2} }}{{2\uppi\tau }}\frac{{\sin \left( {\left( {1 - n_{2} } \right)\uppi} \right)}}{{\cosh \left( {n_{2} \ln \left( {\frac{\tau }{{\tau_{0,2} }}} \right)} \right) - \cos \left( {\left( {1 - n_{2} } \right)\uppi} \right)}}, $$

whilst for synthetic FRAC2 the corresponding DFRT (i.e., DFRT_FRAC2_) is given by:

25 $$ g\left( \tau \right) = \left\{ {\begin{array}{*{20}l} {\frac{{R_{1} }}{\pi }\frac{{\sin \left( {n_{1} \pi } \right)}}{{\tau^{{ - n_{1} }} \left( {\tau_{0,1} - \tau } \right)^{{n_{1} }}  }} + \frac{{R_{2} }}{\pi }\frac{{\sin \left( {n_{2} \pi } \right)}}{{\tau^{{ - n_{2} }} \left( {\tau_{0,2} - \tau } \right)^{{n_{2} }}  }},} \hfill & \quad {{\text{if}}\quad \tau < \tau_{0} } \hfill \\ {0,} \hfill & \quad{{\text{if}}\quad \tau > \tau_{0} .} \hfill \\ \end{array} } \right. $$

### Measured impedance data

For the purpose of this study experimental measurements were performed on solid oxide fuel cells (Fig. 11). The cells were of industrial-size with a chemically active surface of 80 cm^2^, whereby the operating temperature was set to be 800 °C. The fuel electrode was fed with humidified hydrogen, and the air electrode was supplied with air. For the purpose of this study impedance measurements were performed starting with the open circuit conditions, further loading the cell and decreasing the voltage down to 0.7 V. The EIS measurements were carried out using a galvanostatic technique. The AC amplitude was set to be 4% of the DC values, whereby the voltage was measured. The measurements were performed in a frequency range between 10 kHz and 100 mHz. For more detailed information about the experimental setup, the authors refer to Subotić et al. (2018).

### DFRT software used in this work

Although there are several DFRT software that can be used in EIS study (see Sect. 5.1), we decided to apply (and comment^2^) only those listed in Table 2. The first one is FTIKREG developed by Weese (1992), which is a well-known FORTRAN library that has been continuously used to construct DFRT from impedance data. The second one is DRTtools produced by Wan et al. (2015) that applies radial basis functions (RBFs) to discretize DFRT. And the last one, DFRT-Py by Zic and Pereverzyev (2018), which does not apply discretization. Note that programs given in Table 2 are rather different as they apply diverse strategies (e.g., single- and multi-parameter regularization) to reconstruct DFRT data (see details in Sect. 5.1).

**Table 2:** The different strategy utilized in available DFRT softwere that apply regularization

Software	Software requirements (license)	Regularization	Regularization parameter choice	Discretization
FTIKREG	None (free^a^)	Single-parameter	Manual or SC-method (Honerkamp and Weese 1990)	Yes
DRTtools	Proprietary Matlab (free^b^)	Single-parameter	Manual	Yes
DFRT-Py	None (MIT license^c^)	Mulit-parameter	Automatic	None

^a^See http://cpc.cs.qub.ac.uk/licence/licence.html

^b^DRTtools is freely available under the GNU license from this site (https://sites.google.com/site/drttools/)

^c^See https://opensource.org/licenses/MIT

**Fig. 1:**
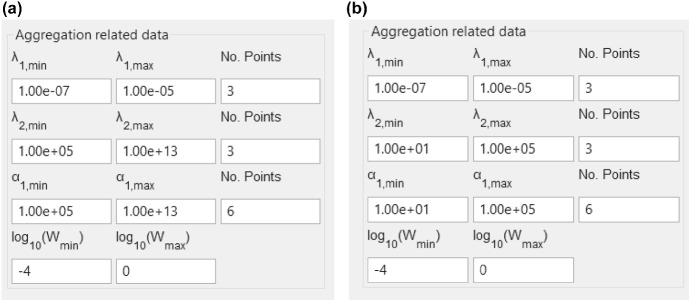
Screenshot of the DFRT-Py regularization parameters that were used in **a** synthetic data study, **b** measured data study. Note that only in the case of measured data study (see **b** inset) the weights (15) were equal to ones

In order to extract DFRT from impedance data by using the listed software (Table 2), one needs to adjust several parameters (Table 3). Specifically, in DRTtools both regularization and RBF shape parameters need to be a priori chosen. However, when chosen they were not modified in this work (unless otherwise specified). Furthermore, the regularization parameter in FTIKREG can be manually given or it can be obtained by using the self-consistent (SC) method (Honerkamp and Weese 1990) (Table 3). And finally, in the case of DFRT-Py, there are two sets of parameters. The first set of parameters (Fig. 1a) was applied in Sects. 5.2–5.4, whilst in Sect. 5.5 we used parameters shown in Fig. 1b.

**Table 3:** Parameters used to extract DFRT from ZARC2 and FRAC2 impedance data in this work

Software	Regularization parameter	Coefficient^a^ to FWHM	Discretization method	Combined $\text{Re} \left( {Z\left( \omega \right)} \right)$ and $\text{Im} \left( {Z\left( \omega \right)} \right)$
FTIKREG	SC-method	None	Gaussian	Yes
DRTtools	10^−3^	0.2	Quadrature	Yes
DFRT-Py	Automatic	None	None	Yes

FWHM: full width at half maximum (RBF shape parameter)

^a^See description in Wan et al. (2015)

As listed programs (Table 2) apply single- and multi-parameter regularization, it is important to emphasize that DFRT data were constructed from combined $\text{Re} \left( {Z\left( \omega \right)} \right)$ and $\text{Im} \left( {Z\left( \omega \right)} \right)$ parts (see Table 3). Interestingly, some researchers prefer the application of only $\text{Re} \left( {Z\left( \omega \right)} \right)$ part because it is less affected by noise and errors (Ivers-Tiffee and Weber 2017). This approach is a common one, especially when dealing with noisy data. On the other hand, there are papers (Dion and Lasia 1999) that claim that better DFRT results are obtained by the usage of only $\text{Im} \left( {Z\left( \omega \right)} \right)$. The choice to use only $\text{Im} \left( {Z\left( \omega \right)} \right)$ can be explained by the fact that DFRT has greater impact on this part of impedance data (see (3)).

## Results and discussion

### Existing DFRT approaches

According to DFRT literature (Ivers-Tiffee and Weber 2017; Kobayashi and Suzuki 2018), there are numerous approaches to extract the Distribution Function of Relaxation Times (DFRT) data from electrochemical impedance spectroscopy (EIS) data. The majority of reported approaches is based on evolutionary programming (Hershkovitz et al. 2011; Tesler et al. 2010) and Monte Carlo techniques (Tuncer and Macdonald 2006), maximum entropy model (Horlin 1998), Fourier filtering (Boukamp 2015; Schichlein et al. 2002), and regularization techniques (Dion and Lasia 1999; Kobayashi et al. 2016; Kobayashi and Suzuki 2018; Wan et al. 2015; Zic and Pereverzyev 2018). Additionally, the first software to extract DFRT from EIS data is based on Fourier transform technique (Kobayashi and Suzuki 2018). However, in this work we are focused on the regularization techniques that are embedded in FTIKREG, DRTtools and DFRT-Py software (Table 2); and thus, there are several facts that should be discussed.

First, the approaches in FTIKREG and DRTtools are based on discretization methods (Table 2). To be precise, in FTIKREG, all functions and operators are approximated by finite-dimensional vectors and matrices (Weese 1992) whereas, in DRTtools the approximation error is somewhat reduced due to the application of radial basis functions (RBFs) (Wan et al. 2015) as discretization basis. On the other hand, DFRT-Py applies table integrals; and thus, any additional discretization errors are avoided (Zic and Pereverzyev 2018). Second, in DRTtools the regularization parameter should be given a priori, whilst in FTIKREG this parameter can be given manually or it can be obtained by a self-consistent (SC) method (Honerkamp and Weese 1990). However, this method is heavily based on the assumption that the noise is independent standard Gaussian random variable (Honerkamp and Weese 1990), which is frequently not the case when dealing with measured EIS data. Oppositely to DRTtools and FTIKREG, in DFRT-Py, the regularized solutions are aggregated, which allows an automatic regularization (Zic and Pereverzyev 2018). Third, the discretization procedure in DRTtools requires a priori choice of RBF shape parameter (Wan et al. 2015), which indicates that both regularization and the shape parameters have to be properly chosen. Interestingly, this action is avoided when operating with FTIKREG and DFRT-Py, as they do not apply any parameterized basis functions. And finally, DRTtools and FTIKREG apply a single-parameter regularization even when using combined real and imaginary impedance parts, whereas DFRT-Py applies a multi-parameters regularization.

To sum up, the aforementioned software (Table 2) apply different approaches to extract DFRT data from the EIS data; and thus, to properly probe different approaches they were tested by a series of the synthetic and measured data.

### DFRT study of noisy ZARC2 and FRAC2 impedance data

**Fig. 2:**
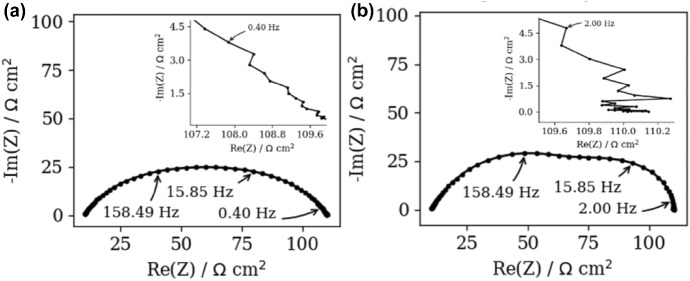
Nyquist spectra of the polluted synthetic, **a** ZARC2, **b** FRAC2 impedance data. Insets in **a**, **b** show the high frequency regions of ZACR2 and FRAC2

To illustrate the impact of the measurements noise, the polluted (*NF* = 0.001) synthetic ZARC2 and FRAC2 data were analyzed (Fig. 2a, b). Nowadays, it is common practice to apply the noisy data in DFRT study and readers are encouraged to review the following papers, e.g., Dion and Lasia (1999) and Wan et al. (2015). Although the application of *NF* = 0.001 offers at least 0.1% of noise (see Eq. (23)), it should be mentioned that normally distributed noise can take arbitrary large values. In this work, the impact of noise can be observed at *f* > 0.4 Hz and *f* > 2.00 Hz (see insets in Fig. 2a, b).

**Fig. 3:**
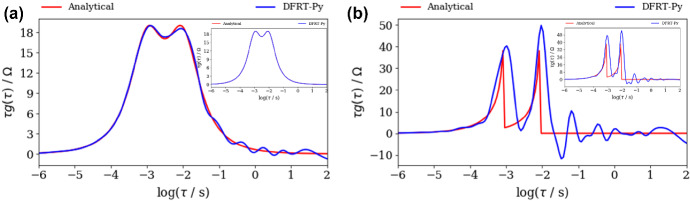
Analytical and aggregated DFRT data of polluted (NF = 0.001), **a** ZARC2, **b** FRAC2 data (Fig. 2a, b) obtained by DFRT-Py. Insets in **a**, **b** show DFRT data of unpolluted ZARC2 and FRAC2

Next, DFRT of ZARC2 and FRAC2 data (Fig. 2a, b) obtained by DFRT-Py software are displayed in Fig. 3. Note that the impact of noise on DFRT_ZARC2_ and DFRT_FRAC2_ data is presented in Fig. 3a, b, whilst insets in Fig. 3a, b represent data obtained from non-polluted synthetic data. The constructed DFRT_ZARC2_ data perfectly match the analytical ones (see Fig. 3a inset), whereas the height of the right (vs. left) DFRT_FRAC2_ peak is somewhat higher. According to Fig. 3a and inset in Fig. 3a, it follows that the noise induced DFRT_ZARC2_ oscillations at *τ* > 0.05 s (Fig. 3a). On the other hand, it is obvious that discontinuities induced DFRT_FRAC2_ oscillations in Fig. 3b inset, whereas these oscillations are additionally amplified due to the presence of noise (Fig. 3b).

**Fig. 4:**
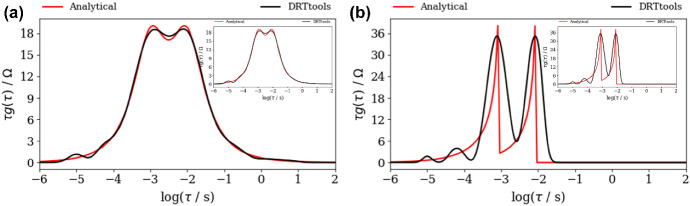
DFRT data of noisy (NF = 0.001), **a** ZARC2, **b** FRAC2 data (Fig. 2a, b) obtained by the DRTtools. Insets in **a**, **b** show DFRT data of unpolluted ZARC2 and FRAC2 data

At the same time, DFRT_ZARC2_ and DFRT_FRAC2_ obtained by DRTtools show no oscillations (Fig. 4a, b). Furthermore, the lack of oscillations can be attributed to the application of RBF (Wan et al. 2015) as a basis for discretization. However, it appears that application of RBF smooths DFRT data that can result in a possible loss of DFRT-related information. Furthermore, the application of DRTtools (Fig. 4a, b) leads to the occurrence of additional border peaks between *τ* = 10^−6^ and 10^−4^ s. The origin of the border peaks in DFRT data (obtained by using FTIKREG) was also discussed in Ivers-Tiffee and Weber (2017). It was concluded that these peaks contain no additional information and that they could be attributed to the presence of the noise. As DFRT-Py yielded data without the border peaks (Fig. 3), it is fair to say that discretization errors in DRTtools might be responsible for the formation of the border peaks in Fig. 4.

### Effect of missing data points on DFRT study

**Fig. 5:**
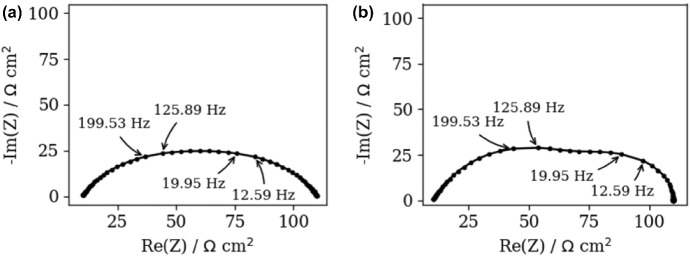
Nyquist spectra of the polluted synthetic, **a** ZARC2, **b** FRAC2 data. The data points related to 15.85 and 158.49 Hz are missing

**Fig. 6:**
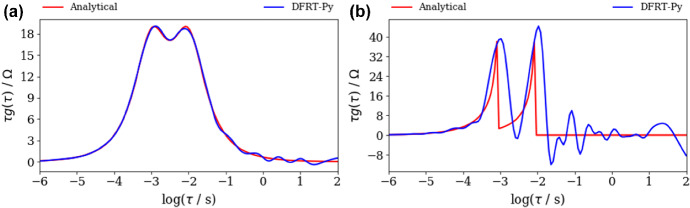
Analytical and aggregated DFRT data of noisy (NF = 0.001), **a** ZARC2, **b** FRAC2 data (Fig. 5a, b) obtained by DFRT-Py

To investigate further the difference between single- and multi-parameters regularization approaches (Table 2), they have been probed by analyzing noisy ZARC2 and FRAC2 data from which two impedance data points at 15.85 and 158.49 Hz are removed (Fig. 5a, b). This frequency values are chosen (Fig. 5) as they correspond to the positions of DFRT_ZARC2_ and DFRT_FRAC2_ peaks maxima (see Fig. 6).

The idea to study the missing data effect originates from the literature (Boukamp and Rolle 2017; Ivers-Tiffee and Weber 2017) as some authors (Boukamp and Rolle 2017) reported that this effect induces changes in DFRT spectra. On the other hand, one group of authors concluded (Ivers-Tiffee and Weber 2017) that this effect has no impact on DFRT study. However, the conclusions presented in Boukamp and Rolle (2017) and Ivers-Tiffee and Weber (2017) are obtained by using two different software (FTIGREG and DRTtools) that apply single-parameter regularization. Thus, it would be intriguing to see the effect of missing data onto both single- and multi-parameter regularizations techniques.

**Fig. 7:**
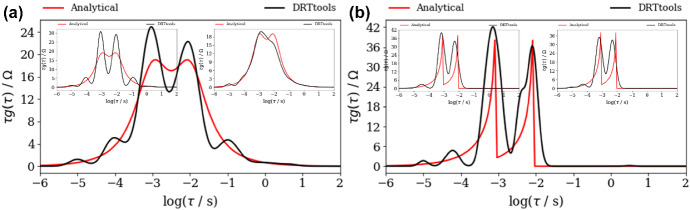
DFRT data of noisy (NF = 0.001), **a** ZARC2, **b** FRAC2 data (Fig. 5a, b) obtained by the DRTtools. Left insets in both **a**, **b** are obtained by using only Re(*Z*(*ω*)) parts, whereas right insets in both **a**, **b** are obtained by using only Im(*Z*(*ω*)) parts

Our experiment shows that when using ZARC2 data (Fig. 5a), DFRT-Py produced two DFRT_ZARC2_ peaks of the same height that perfectly match the analytical ones (Fig. 6a). On the other hand, DRTtools produced two unexpected features in Fig. 7a. The first feature is that DFRT_ZARC2_ peaks are separated by a large depression, which presents a problem because such two peaks configuration can be easily misinterpreted as the one corresponding to DFRT_FRAC2_ peaks (Fig. 7b) i.e., to Davidson–Cole (Davidson and Cole 1951) model but not to Cole and Cole (1941) Cole–Cole model. The second unexpected feature is the fact that there are two additional erroneous peaks at ≈ 10^−4^ and ≈ 10^−1^ s (Fig. 7a), which further hinder DFRT interpretation.

Furthermore, supplementary calculations with DRTtools indicate that when using only real impedance part (left inset in Fig. 7a), both the depression and the peaks turn out to be more pronounced. At the same time, when using only imaginary impedance part (right inset in Fig. 7a), the depression disappears as two DFRT_ZARC2_ peaks are merged into one. This clearly indicates that the *same* regularization parameter value (e.g., 10^−3^) cannot be applied when using single or combined impedance parts in the regularization. To rephrase it, for a proper regularization of combined real and imaginary impedance parts, the multi-parameter regularization (as in DFRT-Py) seems to be unavoidable.

Next, a computation of FRAC2 impedance data shows that both DFRT-Py (Fig. 6b) and DRTtools (Fig. 7b) are not drastically affected by the missing data effect. To be specific, this effect increased oscillation at *τ* > 0.01 s in Fig. 6b, but there are no additional peaks in Fig. 7b. Moreover, it appears that DRTtools yielded almost identical data in Fig. 7b and in Fig. 7b insets, which suggests that the missing data effect is not observed because RBF *cannot* properly mimic discontinuities in DFRT_FRAC2_ anyway.

To summarize, it appears that both the missing data effect and the application of additional discretization basis (i.e., RBFs) yield similar DFRT_ZARC2_ and DFRT_FRAC2_ pictures and hinder their distinguishment. At the same time, this kind of problems can be avoided when using DFRT-Py as this software does not apply any unnecessary discretization techniques. However, the full benefit of the application of the regularization without unnecessary discretization will become obvious in the next section when using impedance data corresponding to randomly spaced (in logarithmic scale) frequency values.

### Effect of unequally spaced frequency data on DFRT_ZARC2_ and DFRT_FRAC2_ study

**Fig. 8:**
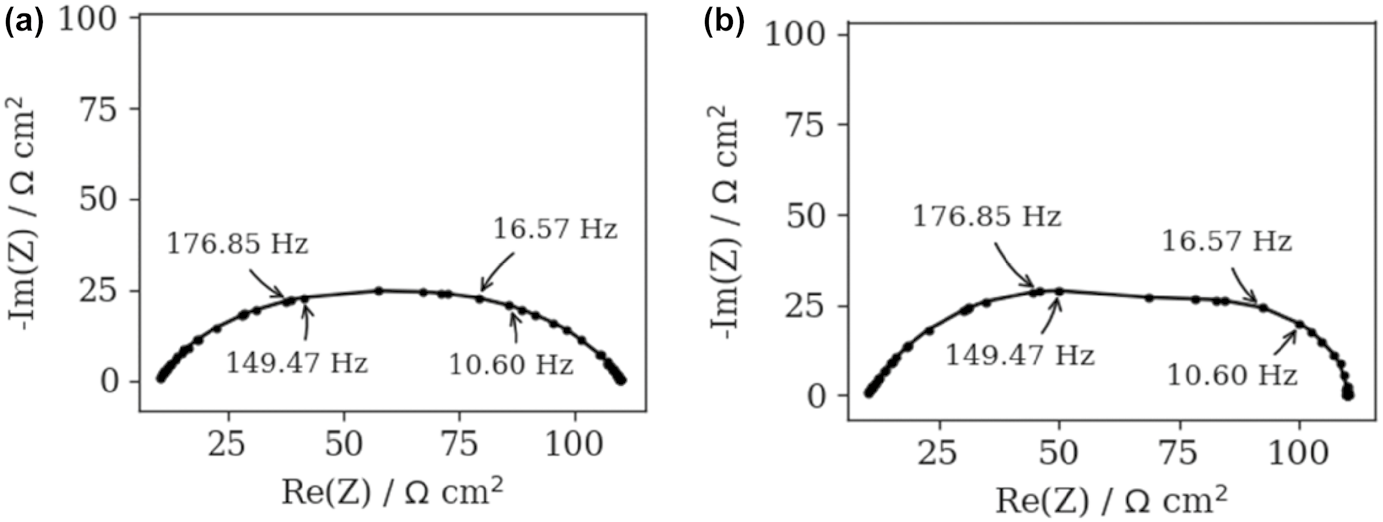
Nyquist spectra of the polluted synthetic, **a** ZARC2, **b** FRAC2 data obtained by using randomly spaced frequency data points

In order to further inspect advantages of avoiding unnecessary discretization (see Table 2), the noisy ZARC2 and FRAC2 data (Fig. 8a, b) are prepared by using unequally (i.e., randomly) spaced frequency values from 0.01 Hz to 100 kHz interval. The data points are also randomly spaced around points at 15.85 and 158.49 Hz that can be related to DFRT peaks. Although such kind of spacing is not so usual in practice, these data (Fig. 8a, b) can be used as, e.g., a dataset for testing newly developed DFRT methods.

**Fig. 9:**
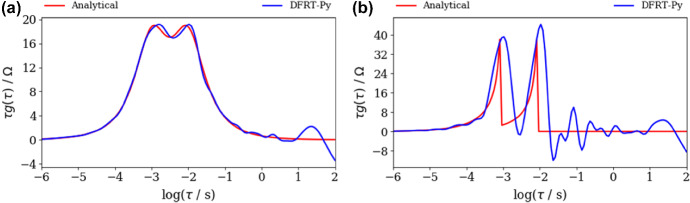
Analytical and aggregated DFRT data of noisy (NF = 0.001), **a** ZARC2, **b** FRAC2 data (Fig. 8a, b) obtained by DFRT-Py

Figure 9a, b display DFRT_ZARC2_ and DFRT_FRAC2_ data approximated by DFRT-Py. The oscillations in Fig. 9a, b are increased, which is attributed to the “higher” level of data corruption (i.e., application of unequally spaced frequency data). The positions of reconstructed (vs. analytical) DFRT_ZARC2_ peaks show insignificant offset towards right, but the peaks are of the same height. Furthermore, the height of the right (vs. left) DFRT_FRAC2_ peak is slightly increased, which can be attributed to the vicinity of two discontinuities. Overall, DFRT-Py yielded DFRT_ZARC2_ and DFRT_FRAC2_ peaks with positions that match well to the positions of the analytical ones.

**Fig. 10:**
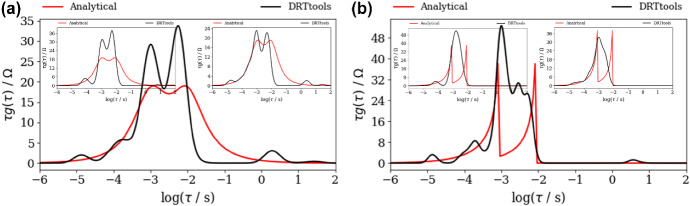
DFRT data of noisy (NF = 0.001), **a** ZARC2, **b** FRAC2 data (Fig. 8a, b) obtained by the DRTtools. Left insets in **a**, **b** are obtained by using only Re(*Z*(*ω*)) parts, whereas right insets in **a**, **b** are obtained by using only Im(*Z*(*ω*)) parts

On the other hand, it appears that DFRT_ZARC2_ and DFRT_FRAC2_ peaks obtained by DRTtools are shifted to the left (Fig. 10a, b). Additionally, DFRT_ZARC2_ peaks are separated by depression, whilst DFRT_FRAC2_ peaks are merged. To be exact, Fig. 10b suggests that the right DFRT_FRAC2_ peak is moved to the left and merged with the left one. The same observation can be obtained by analyzing data in Fig. 10b insets. This indicates that the considered level of data corruption is so “high” that RBF discretization fails in reconstruction.

In contrast, DFRT-Py yielded DFRT peaks with no positions offset, but with higher level of oscillation.

### Real experiment data in DFRT_ZARC2_ and DFRT_FRAC2_ study

**Fig. 11:**
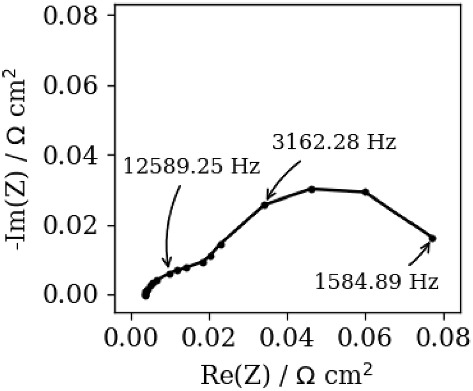
Measured experimental impedance data of industrial-sized solid oxide fuel cells

The next experiments uses measurements of Nyquist spectra of industrial-size oxide fuel cell displayed in Fig. 11. By comparing this figure with Fig. 8, one may conclude that the level of data perturbation in Fig. 11 is similar to that in Fig. 8. Moreover, Fig. 11 hints that the data are characterized by two time constants that should yield two DFRT peaks. Furthermore, the number of frequency data points is 19, which is much less (< 71) than in the case of synthetic data experiments (Sects. 5.2–5.4). However, a low number of data points is desirable as it reduces total measurement time, which enables fast insight in DFRT data.

**Fig. 12:**
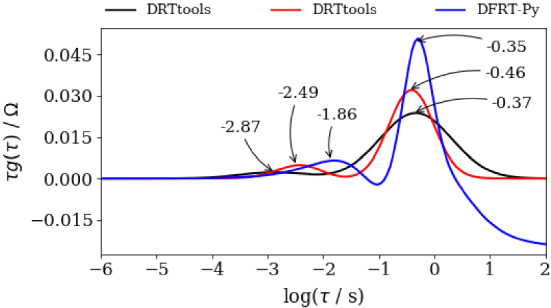
DFRT data of measured impedance experimental data obtained by DFRT-Py and by DRTtools. Symbol reference: black (–) and red (–) curves were collected when DRTtools applied two different shape factors (0.2 and 0.35; see Table 3), whilst blue (–) curve was obtained by DFRT-Py (color figure online)

Figure 12 represents DFRT curves obtained by DRTtools and DFRT-Py. DRTtools data were obtained by the application of two different values of FWHM coefficients (see Table 3), namely 0.2 and 0.35, whilst DFRT-Py parameters are shown in the screenshot b) of Fig. 1. DFRT curves in Fig. 12 are characterized by two peaks, i.e., by one modest and one prominent. It appears that the positions of the reconstructed prominent DFRT peaks are rather similar (i.e., − 0.35, − 0.37, and − 0.46). At the same time, the positions of the modest DFRT peaks exhibit an excessive offset (− 2.87, − 2.49, and − 1.86). Recall that similar offset in the peaks’ positions was detected in Fig. 10, which was attributed to high level of impedance data perturbation (Fig. 8). Therefore, according to Fig. 12, it is fair to conclude that when data quality and number of data points are low both software should be used side by side in DFRT study.

## Conclusions

We have tested and analyzed some of the available DFRT programs based on different regularization strategies i.e., FTIKREG and DRTtools apply single-parameter regularization and diverse discretization techniques whereas, DFRT-Py applies the multi-parameter regularization without any additional discretization.

Our tests show that a single-parameter regularization is suitable for moderately corrupted impedance data. On the other hand, a multi-parameter regularization approach is able to handle the cases where the level of data corruption is higher.

In this work, the positions of reconstructed DFRT_ZARC2_ and DFRT_FRAC2_ peaks were always equal to the analytical ones only in the case of DFRT-Py. This clearly supports our belief that a full regularization effect can only be obtained when using multi-parameters regularization and directly applying it to impedance data without any additional discretization.

However, when low quality measured experimental data were analyzed by methods under comparison, the positions of DFRT peaks were not the same. This indicates that both software should be used when dealing with low quality data.

## Footnotes

1 This step allows us to avoid additional discretization; and thus, no numerical integration error will occur.

2 FTIKREG was not used herein, but its results from literature were commented on.
